# Auto-brewery syndrome with Candida-associated gastric ulcers in a severely cachectic child

**DOI:** 10.1007/s12024-025-01170-w

**Published:** 2026-01-12

**Authors:** Silvia Farkašová Iannaccone, Alžbeta Ginelliová, Viktória Briškárová, Daniel Farkaš

**Affiliations:** 1 Department of Forensic Medicine, Faculty of Medicine, Pavol Jozef Šafárik University, Trieda SNP 1, 040 11, Košice, 040 11 Slovakia; 2 Medicolegal Department of Healthcare Surveillance Authority, Ipeľská 1, Košice, 043 74 Slovakia

**Keywords:** Gastric ulcer, Candida krusei, Pichia kudriavzevii, Endogenous ethanol fermentation, Auto-brewery syndrome

## Abstract

Auto-brewery syndrome is a rare medical phenomenon caused by fungal gut dysbiosis in which ethanol is produced through endogenous fermentation by fungi or bacteria in the gastrointestinal system. The clinical manifestations of auto-brewery syndrome are related to the blood alcohol level concentration. A 16-year-old severely disabled cachectic boy with congenital brain malformations and cerebral palsy experienced substantial weight loss following COVID-19 infection 6 months before his death. He did not receive the COVID-19 vaccine. He was repeatedly treated for pneumonia, most recently a week before his death. He was found by his parents experiencing breathing difficulty and he could not be resuscitated. The autopsy revealed severe cachexia and gastric ulcers with fungal infiltration. The bacteria and Candida krusei detected through microbiological testing showed identical pattern as the fungi found in the stomach. Toxicological analysis using gas chromatography revealed a blood alcohol level of 0.75 g/L. Death was attributed to pneumonia due to depletion of body reserves. A high peroral sugar intake in individuals with specific medical conditions (such as malnutrition, cachexia) may result in endogenous alcohol production when fermenting yeast or bacteria in the gastrointestinal tract becomes pathogenic, causing auto-brewery syndrome. Failure to perform microbial testing of the gastric and intestinal contents can result in incorrect interpretation of toxicological results. The incorrect interpretation of ethanol in the blood of a child (especially with severe disabilities) without considering auto-brewery syndrome can have serious medical, social and legal consequences. *Foetor ex ore* and signs of alcohol intoxication (such as poor coordination, slurred speech, impaired vision, etc.) with no history of alcohol consumption in disabled individuals with a history of high-sugar diet should prompt a clinician to suspect auto-brewery syndrome.

## Key points

A high peroral sugar intake in individuals with specific medical conditions may result in endogenous alcohol production.

Auto-brewery syndrome is a rare medical phenomenon caused by fungal gut dysbiosis in which ethanol is produced through endogenous fermentation.

The incorrect interpretation of ethanol in the blood without considering auto-brewery syndrome may have serious medical and legal consequences.

Signs of alcohol intoxication with no history of alcohol consumption in disabled individuals with a history of high-sugar diet should prompt a clinician to suspect auto-brewery syndrome.

## Background

Candida is a fungus that commonly colonizes the gastrointestinal tract of healthy individuals; however, higher level of Candida colonization is associated with diseases of the gastrointestinal tract [1] in both immunocompetent and immunocompromised patients [2]. In health care facilities, Candida remains a major cause of nosocomial infections, frequently causing fungemia [3]. The emergence of antifungal resistance among Candida species is a major concern [4]. Gastric candidiasis causing perforation and peritonitis is rare [5]. Duodenal and jejunal candidiasis are uncommon entities [6]. Cases of gastric, esophageal, and duodenal candidiasis with mucosal ulcerations have been rarely described in the literature [7]. Candida krusei is an emerging nosocomial fungal pathogen characterized by a wide spectrum of clinical manifestations and intrinsic resistance to fluconazole [8]. Candida krusei can be associated with gastritis, gastric ulcer, hemorrhage [1]. Some patients may experience extra-intestinal manifestations, such as leg ulcers [9]. The mortality rate of Candida krusei is lower in pediatric compared to adult populations, which may be due to the severity of comorbidities in adults. The risk factors of infection by Candida krusei involve prior antibiotic use [10]. Genetic testing has revealed that the opportunistic pathogen responsible for yeast infections, Candida krusei, and the industrial yeast Pichia kudriavzevii are identical [11]. Pichia kudriavzevii, Saccharomyces krusei, and Issatchenkia orientalis are synonymous names for the same yeast and continue to be used in the medical literature [12].

Auto-brewery syndrome (also known as ABS, gut fermentation syndrome, endogenous ethanol fermentation or drunkenness disease) is a rare medical phenomenon caused by fungal gut dysbiosis in which ethanol is produced through endogenous fermentation and may mimic a food allergy or intolerance [13]. ABS is linked to short bowel syndrome [14, 15], laparotomy, gastrectomy [16], Crohn disease, high sugar intake [17], and antibiotic treatment [18]. In cases of ABS, evaluation should include a detailed history regarding diet and alcohol intake, laboratory test including metabolic panel, blood alcohol level, drug screen, microbial testing and a positive glucose challenge test with blood ethanol levels rising 0.5 mg/dL or more after a glucose tolerance test to confirm endogenous alcohol production [19]. The diagnosis of ABS can be challenging. A thorough medical history regarding diet and current medications, signs of alcohol intoxication without a history of alcohol consumption, laboratory testing including blood alcohol levels and a glucose challenge test may be helpful. Treatment depends on the underlying cause of ABS and may include diet modification, avoiding alcohol consumption and antifungal or antibiotic therapy depending on the causative agent [15, 17, 18].

## Case presentation

**Fig. 1:**
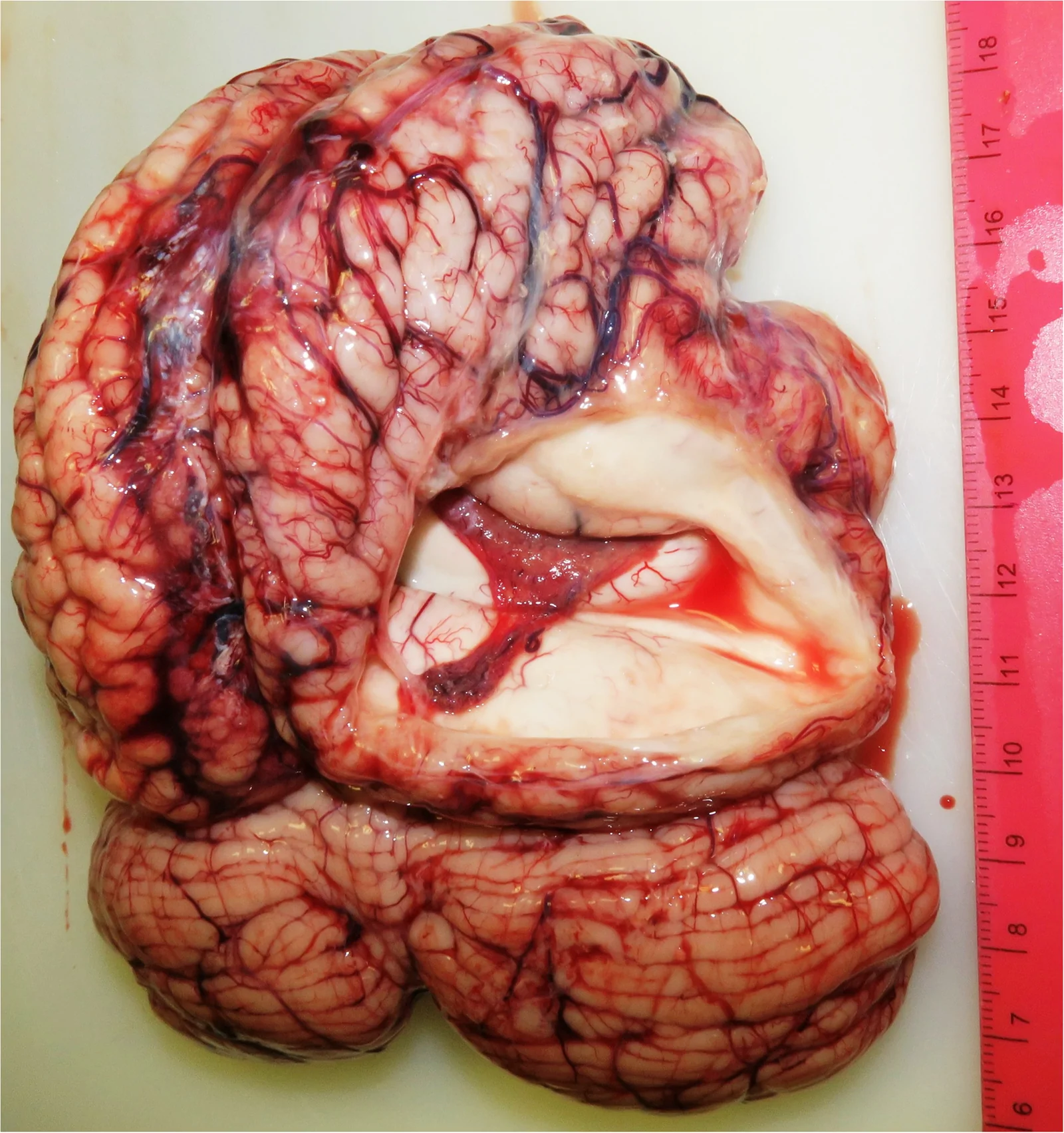
Gross photograph of the brain showing a prominent schizencephalic cleft of the right temporal lobe

**Fig. 2:**
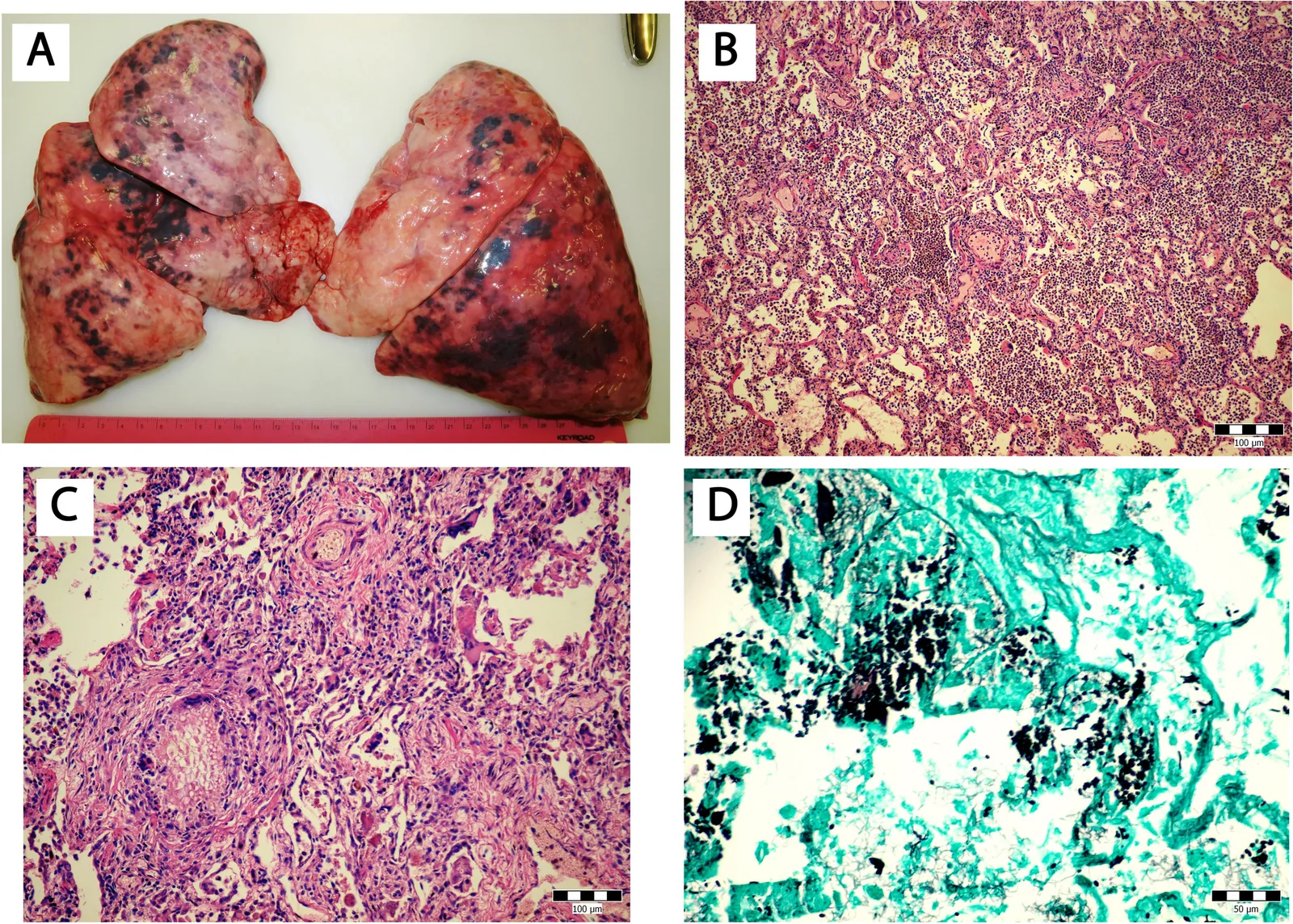
(**a**) Gross photograph of the lungs showing poorly-demarcated areas of coffee ground-like gastric contents aspiration. (**b**) Confluent pneumonia (hematoxylin-eosin 600x). (**c**) Granulomatous reaction following chronic aspiration (hematoxylin-eosin 200x). (d) Grocott silver stain of Candida yeast in the alveoli (400x)

**Fig. 3:**
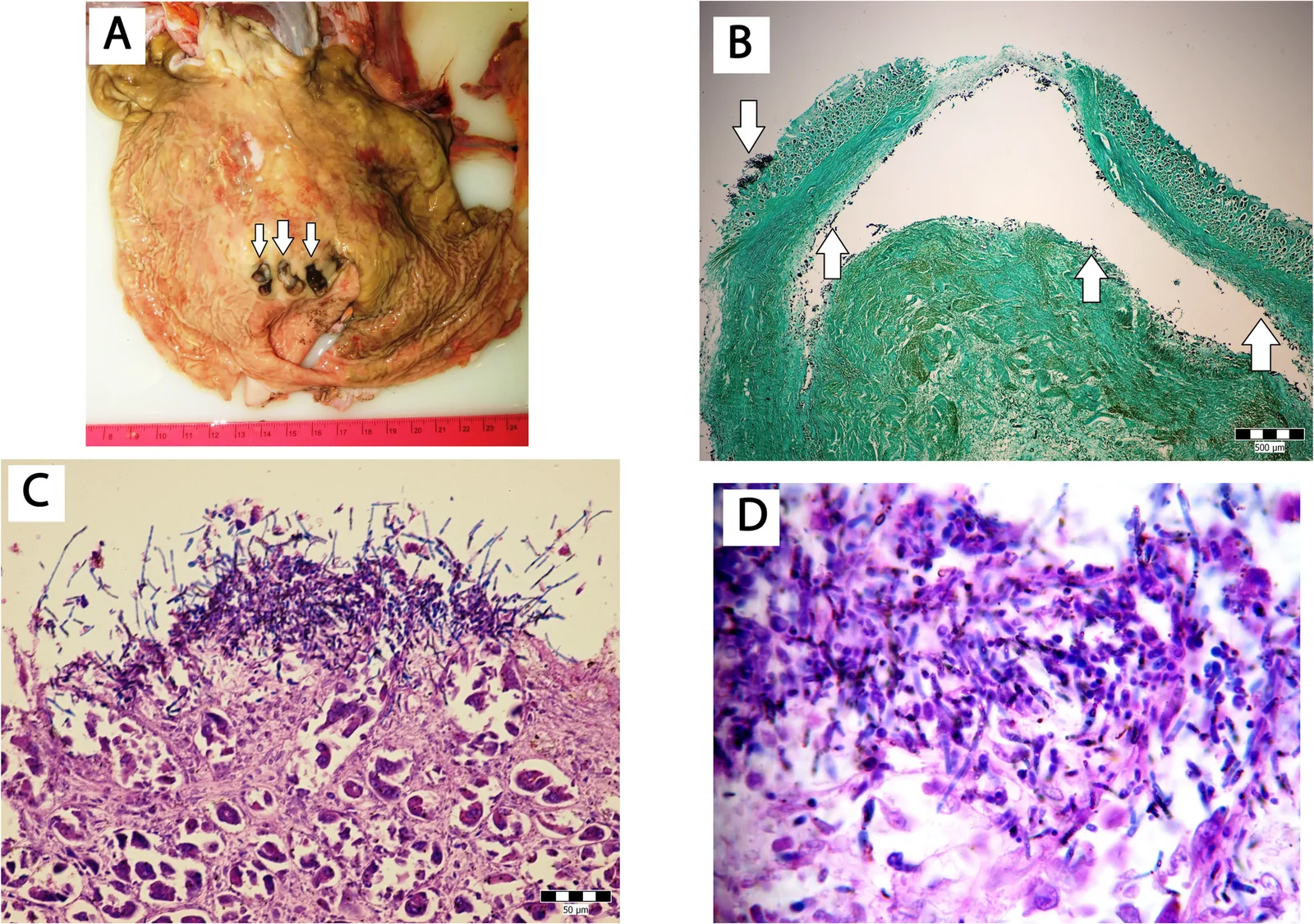
(**a**) Three isolated ulcers (arrows) of the gastric mucosa. (**b**) Gastric ulcer with Candida colonization of the mucosa (down arrow) and at the site of the mucosal detachment (up arrows) /Grocott silver stain 40x/. (**c**) Invasive candidiasis of the gastric mucosa (hematoxylin-eosin 400x). (**d**) High magnification micrograph showing Candida yeast (hematoxylin-eosin 1000x)

A 16-year-old polymorbid boy with congenital brain malformations (schizencephaly, pachygyria), cerebral palsy with spastic quadriplegia, severe mental impairment, scoliosis, and limb deformities is described in this case report. The patient had been treated for epilepsy since he was three months old. Due to a high-arched palate and dysphagia, he could only ingest mixed and liquid food. He was never treated surgically (e.g. esophagectomy or gastrectomy). He experienced substantial weight loss (12 kg, a third of his initial weight) following COVID-19 infection 6 months before his death. Over the past few months, he had reduced appetite and recurring pneumonia with high fever. A week before his death, he was treated with antibiotics for pneumonia. During his life, he had no symptoms of gastrointestinal bleeding, and he was never prescribed immunosuppressants, acid suppressants or nonsteroidal anti-inflammatory drugs. At the time of his death, he was found by his parents experiencing breathing difficulty and dyspnea. Upon arrival of the emergency medical services, the patient showed signs of aspiration of a significant amount of black liquid gastric content, which required airway suctioning. Attempts at resuscitation were unsuccessful. An autopsy was conducted 10 h after the patient’s death. At autopsy the body was that of an extremely cachectic young male (length: 160 cm; weight: 21 kg; body mass index: 8.2) with scoliosis and limb deformities. The brain was abnormally small, weighting only 405 g (microcephaly). The right temporal lobe showed an irregular defect with smooth edges measuring 5 × 5 cm – a prominent cleft into the cerebral cortex (schizencephaly) (Fig. 1). The cleft displayed a deep visible channel into the ventricle. The ventricles showed a mild bilateral enlargement. Marked deformities of the chest and abdominal walls and organ shrinkage were observed. The lungs appeared heavy and consolidated (Fig. 2a). Multiple poorly-demarcated dark brown areas resembling the aspirated gastric content were observed on the cut surface of the lungs. Foci of purulent inflammation were scattered throughout the lungs. The significantly smaller heart had a normal configuration. The stomach contained approximately 15 ml of dark brown fluid. In the prepyloric region of the stomach, there were three isolated defects with dark brown edges embedded in the surrounding mucosa measuring 15 × 7 mm (Fig. 3a). Histologic examination revealed focal fungal colonization of the stomach, including penetration through the surface epithelium into the submucosa (Fig. 3c). Areas of gastric mucosal ulcerations exposing the muscle layer and exhibiting detachment of the mucosal edges were observed, with fungi present at the ulcer base (Fig. 3b) and purulent inflammation reaching the serosa. Fungal angioinvasion was not present. The fungi (spherical, oval, and hyphae) were periodic acid-Schiff (PAS), Grocott, and Gram positive, and some showed the presence of budding (Fig. 3d). Areas of purulent pneumonia (Fig. 2b) and sporadic granulomas with foreign body giant cells following aspiration, were observed (Fig. 2c). The most striking finding in the Grocott (Fig. 2d) and Gram stained sections of the lung was aspiration of digested blood from the stomach containing bacteria and fungi, which showed identical pattern as the fungi found in the stomach. The fungi were found to be near the digested erythrocytes. Microscopic examination of the schizencephalic cleft showed lack of normal lamination and dysplastic abnormal cortex with no sign of inflammation. The liver showed brown atrophy. The iliopsoas muscle demonstrated atrophy with mild fibrosis. No microscopic abnormalities were identified in the histologic sections of the heart, kidney, pancreas, and thyroid gland. Toxicological analysis using gas chromatography revealed a blood alcohol level of 0.75 g/L and a urine alcohol level of 0.01 g/L with no putrefactive products. A urine drug screen detected carbamazepine metabolites. Klebsiella pneumoniae, Enterobacter hormaechei (bacteria) and Pichia kudriavzevii (yeast) were detected through microbiological testing using MALDI-TOF (Matrix Assisted Laser Desorption/Ionization – Time of Flight). After a thorough investigation of the circumstances leading up to and surrounding the death, a complete autopsy, a microbiological testing, and a toxicology analysis, death was attributed to pneumonia due to depletion of body reserves. Further information obtained from the parents revealed that in addition to his breakfast, snack, lunch, and dinner of mixed food, the boy ingested an additional teaspoon of honey and at least 100 ml of apple juice (containing at least 20 g of sugar) six hours before his death. This amount of ingested sugar could have led to the alcohol level detected in the blood of the deceased produced through endogenous fermentation within the digestive system. The diagnosis of ABS was made based on a thorough and conclusive review of all the facts and information concerning the circumstances of the death, in conjunction with the findings at autopsy and the laboratory tests.

## Conclusions

Schizencephaly is a rare congenital brain malformation characterized by abnormal clefts lined with heterotopic gray matter that form the ependyma of the cerebral ventricles to the pia mater. It is a cell migration defect, which develops between the second and the fifth months of gestation [20]. Schizencephaly can be unilateral or bilateral [21, 22], open or closed lip, depending on whether the clefts communicate with the ventricles [23], or associated with epilepsy [24]. Symptoms can vary widely depending on the location of the clefts. Schizencephaly can be associated with late onset psychiatric conditions [25]. In this case, the deceased had unilateral open lip schizencephaly affecting the right temporal lobe with motor deficit, intellectual impairment and epilepsy diagnosed at the age of 3 months. He had been taking antiepileptic drugs since he was diagnosed with epilepsy. At autopsy, trace amount of carbamazepine was detected in his urine. The patient was not immunocompromised, but due to his medical condition, he was not recommended to receive the COVID-19 vaccine. He lost 12 kg – a third of his weight, following a severe COVID-19 infection 6 months before his death. After the COVID-19 infection, the patient was repeatedly treated for pneumonia, weakness, and fatigue – common symptoms of long COVID [26]. Long COVID has a negative impact on the body systems, including the skeletal muscles [27], observed in critically ill patients [28], as in this case.

Candida albicans is the most common Candida species colonizing the gastrointestinal [29]; the esophagus being affected more often than the stomach [30]. We described a case of gastric candidiasis with mucosal ulcerations, without vascular invasion (vasculitis), and no sign of esophageal involvement. Microscopic examination of the stomach and the lungs revealed the presence of fungi with identical histologic features in both organs. The fungal-specific stains (Grocott silver, PAS) highlighted yeasts with septate hyphae, dichotomous branching, and yeast cells. The unusual gross appearance of the lungs required microbiological examination. The presence of fungi in the stomach was not initially considered, therefore, the stomach tissue was not submitted for microbiological testing. Candida species were identified in the lungs with conventional testing, followed by MALDI-TOF identification of Candida krusei (Pichia kudriavzevii). The intestinal mucosa appeared to be unremarkable and was not submitted for microbiological testing.

ABS may have serious medical and legal consequences. It has been identified in both male and female adults and children [18] and it can be caused by common yeasts such as Saccharomyces cerevisiae [15], Saccharomyces boulardii, Candida albicans, Candida krusei, Candida glabrata [13], or bacteria such as Klebsiella pneumoniae [31], Enterococcus faecium [13]. It is highly probable that agonal aspiration of gastric contents with yeasts microscopically detected at the ulcer base resulted in Candida krusei detection in the lungs through microbiological testing. The clinical manifestations of ABS are related to the blood alcohol level concentration. A blood alcohol level as high as 3.5 g/L in a child has been described in the literature [14]. In our case, the child had a blood alcohol level of 0.75 g/L at autopsy. Due to communication difficulties associated with severe mental disability, the parents did not register any behavioral changes.

This case is a rare presentation of ABS in an immunocompetent patient with invasive gastric candidiasis. Long COVID-19, severe cachexia, repeated courses of antibiotic therapy and a high-sugar diet may have contributed to the pathogenesis of ABS.

## Data Availability

Not applicable.

## Abbreviations

ABS: Auto-brewery syndrome
MALDI-TOF: Matrix assisted laser desorption/ionization – time of flight
PAS: Periodic acid-Schiff
